# The potential impact on obesity of a 10% tax on sugar-sweetened beverages in Ireland, an effect assessment modelling study

**DOI:** 10.1186/1471-2458-13-860

**Published:** 2013-09-17

**Authors:** Adam DM Briggs, Oliver T Mytton, David Madden, Donal O’Shea, Mike Rayner, Peter Scarborough

**Affiliations:** 1 British Heart Foundation Health Promotion Research Group, Nuffield Department of Population Health, University of Oxford, Oxford, UK; 2 UK Health Forum, London, UK; 3 School of Economics, University College Dublin, Dublin, Ireland; 4 Department of Endocrinology, St Columcille’s Hospital, Health Service Executive, Loughlinstown, Ireland

**Keywords:** Taxation, Obesity, Overweight, Nutrition, Sugar-sweetened beverages

## Abstract

**Background:**

Some governments have recently shown a willingness to introduce taxes on unhealthy foods and drinks. In 2011, the Irish Minister for Health proposed a 10% tax on sugar sweetened beverages (SSBs) as a measure to combat childhood obesity. Whilst this proposed tax received considerable support, the Irish Department of Finance requested a Health Impact Assessment of this measure. As part of this assessment we set out to model the impact on obesity.

**Methods:**

We used price elasticity estimates to calculate the effect of a 10% SSB tax on SSB consumption. SSBs were assumed to have an own-price elasticity of −0.9 and we assumed a tax pass-on rate to consumers of 90%. Baseline SSB consumption and obesity prevalence, by age, sex and income-group, for Ireland were taken from the 2007 Survey on Lifestyle and Attitude to Nutrition. A comparative risk assessment model was used to estimate the effect on obesity arising from the predicted change in calorie consumption, both for the whole population and for sub-groups (age, sex, income). Sensitivity analyses were conducted on price-elasticity estimates and tax pass-on rates.

**Results:**

We estimate that a 10% tax on SSBs will result in a mean reduction in energy intake of 2.1 kcal/person/day. After adjustment for self-reported data, the 10% tax is predicted to reduce the percentage of the obese adult population (body mass index [BMI] ≥30 kg/m^2^) by 1.3%, equating to 9,900 adults (95% credible intervals: 7,750 to 12,940), and the overweight or obese population (BMI ≥ 25 kg/m^2^) by 0.7%, or 14,380 adults (9,790 to 17,820). Reductions in obesity are similar for men (1.2%) and women (1.3%), and similar for each income group (between 1.1% and 1.4% across income groups). Reductions in obesity are greater in young adults than older adults (e.g. 2.9% in adults aged 18–24 years vs 0.6% in adults aged 65 years and over).

**Conclusions:**

This study suggests that a tax on SSBs in Ireland would have a small but meaningful effect on obesity. While such a tax would be perceived as affecting the whole population, from a health prospective the tax will predominantly affect younger adults who are the main consumers of SSBs.

## Background

In the past two years, governments in Europe and elsewhere have shown a willingness to introduce taxes on unhealthy foods [1,2]. This appears to have been prompted by growing concern about the rising cost of non-communicable disease, particularly that related to obesity, and a need to raise revenue. The marketing and availability of unhealthy foods is increasingly recognised as an important determinant of food consumption and health [3]. This may provide a justification for government intervention, which is increasingly seen as necessary to address the rising burden of non-communicable diseases [4].

Sugar sweetened beverages may be a sensible target for such a tax. Consumption of SSBs is associated with obesity, dental caries and type-2 diabetes [5-7]. Recent trial evidence found that substitution of SSBs with diet drinks among children was associated with lower weight gain [8,9]. France introduced a tax on soft drinks (including diet drinks) in January 2012 [2]. An SSB tax was proposed as part of US healthcare reform in 2009, but was not enacted in the face of heavy industry lobbying [10]. Interest in the idea has also been growing in the UK since the Prime Minster, David Cameron, declared that taxes on unhealthy food is something that should be looked at [11-14]. SSBs have also been a focus for other types of regulation, such as restrictions on portion sizes [15].

Historically, Ireland has taken a proactive stance towards its public health. In 2004, it was the first European country to introduce smoke free workplaces, [16] and has recently proposed a ban on the sponsorship of sports events by the alcohol industry [17]. In 2011 the Health Minister for Ireland, Dr James Reilly, announced that the government was considering the idea of a tax on SSBs [18]. This followed concern about the growing burden of overweight and obesity in Ireland, particularly among the young. Child overweight and obesity has increased significantly. Now a quarter of children aged nine years of age are overweight or obese (19% overweight and 7% obese) [19]. Adult overweight and obesity together account for 61% of the population (37% overweight and 24% obese) [20]. The rising diabetes prevalence is also a concern. Diabetes has increased from 3.4% in 2003 to 6.1% in 2011 [21,22].

The idea of a special tax was considered by the Irish Government’s Special Action Group on Obesity, who established an independent scientific Steering Group that undertook a Health Impact Assessment on a proposed tax of 10% on SSBs in Ireland. Following the Health Impact Assessment, the Special Action Group on Obesity met the Health Minister for Ireland and made a strong proposal to recommend a 10% tax on SSBs on health grounds. The Health Minister for Ireland then wrote to the Finance Minister requesting that this measure be introduced in the budget, however the Finance Minister did not support the proposal, at that time, as SSBs were already subject to a high level of taxation [23]. The proposed excise tax would have been additional to the 23% value added tax (VAT) that is currently levied on SSBs in Ireland (with staple foods being exempt from VAT) [24]. As part of the Health Impact Assessment we were invited to model the effects of a 10% tax on SSBs in Ireland, and it is these results that we present here [25].

## Methods

**Figure 1:**
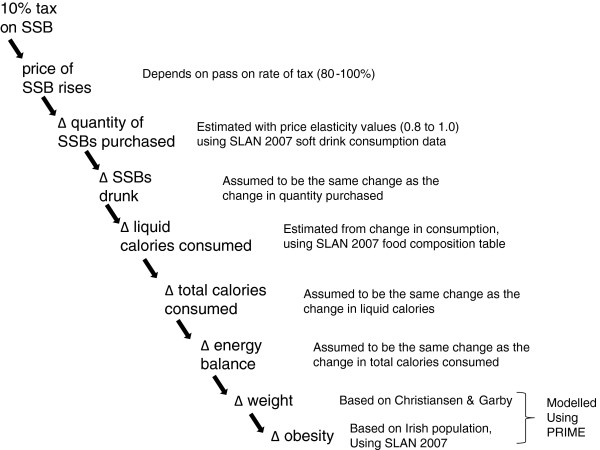
Assumed causal pathway for the effect of a 10% SSB tax on obesity in Ireland.

We used price elasticity estimates to calculate the effect of a 10% SSB tax on SSB purchases and consumption. From this, the change in energy intake was derived. We then used estimates of the change in energy intake to model the effect on body weight and obesity. This approach has been used by others to estimate the effects of a tax on SSBs on obesity [26-28]. The modelled causal pathway is set out in Figure 1.

### Data and assumptions

#### Price elasticities

Price elasticity measures the responsiveness of purchasing of a good when prices change. Own price values refer to the change in purchases that occur for a given good when the price of that same good alters. Cross-price values refer to the change in purchases that occur for a given good when the price of another good alters (e.g. the purchases of cream when the price of strawberries changes).

SSBs for each age and income group were assumed to have an own-price elasticity of −0.9 (i.e. a 10% price rise causes consumption to fall by 9%). This estimate was conservatively based on two studies. Firstly, a systematic review of US food price elasticity data found that the mean price elasticity of demand for soft beverages was −0.79 (range −0.13 to −3.18), averaged across 14 studies [29]. Secondly, Ireland had a special excise tax on soft drinks (both diet and non-diet drinks) in the 1980s, from which it was estimated that the price elasticity of demand for soft drinks in Ireland as a result of this tax was −1.10 [30]. This estimate agrees well with a recent meta-analysis estimate of −0.93 [31]. Cross-price effects were not modelled as we were unable to identify any Ireland specific cross-price elasticities. It was not felt appropriate to use available estimates from other countries (e.g. the US) as the values are likely to be context specific.

#### Pass on rate to consumers

The pass on rate of the tax to consumers may be less than, equal to, or greater than 100%. While theory suggests this may be predicted by the type of competition that exists within the market and other market factors (e.g. marginal cost), in practice this is often not possible to estimate and empirical evidence may provide the best clues. Empirical studies are limited and provide a mixed picture. For example data from the USA suggest a pass on-rate in excess of 100% for soft drinks [32]. However the study of the Irish tax on SSBs in the 1980s suggests that the pass on rate was less than 100% (meaning that manufacturers and retailers absorb some of the tax increase by reducing profit margins), but this study did not quantify the magnitude of the pass on [30]. Where there is a degree of uncertainty with the pass-on rate, in the UK context it is considered reasonable to assume a pass on rate of 100% [33]. For these reasons we took a conservative range of 80 to 100%, which we thought to be reasonable in the Irish context. Since our original work, results from France on the pass on rate have been published. This found a pass-on rate of 100% for soda drinks, 60% for fruit drinks and 85% for flavoured waters [34]. We also note that alcohol taxes in the US tend to be passed on fully [35]. This appears consistent with our pass on rate of 90% (range 80 to 100%). A 90% pass on rate means that 90% of the 10% tax is passed on to the consumer (i.e. a 10% tax would generate a 9% price rise).

#### Sugar-sweetened beverages consumption in Ireland

The Survey on Lifestyle and Attitude to Nutrition (SLAN) periodically collects lifestyle and nutrition data on over 10,000 adults living in Ireland. The 2007 survey used face-to-face interviews to collect information on health behaviour; the 2007 dataset is representative of the Irish population [20]. The SLAN dataset was chosen in preference to a comparable dataset the National Adult Nutrition Survey 2011 conducted by the Irish Universities Nutrition Alliance (IUNA), because it is much larger (IUNA survey 1,500 adults compared to 10,364 for SLAN, 2007). Using the SLAN dataset brought greater precision to estimates by sub-groups (age, sex and income).

The SLAN dataset was used to estimate SSB consumption, as well as obesity prevalence for Ireland [20]. SSBs were defined as soft drinks with added sugar, comprising of two categories from the SLAN dataset, “Fizzy soft drinks” (not low calorie or diet) and “Fruit squash”. An adapted version of the Willet food frequency questionnaire, [36] part of the SLAN survey, was used to estimate consumption of SSBs by age, sex, and income group (using self-reported consumption of carbonated and non-carbonated SSBs).

#### Overweight and obesity in Ireland

Estimates for overweight and obesity prevalence were taken from the SLAN dataset. Self-reported body mass index (BMI - calculated from self-reported height and self-reported weight) was available for nearly all respondents in the SLAN dataset. Measured BMI (based on objectively measured height and objectively measured weight) was only available for a sub-sample (n = 2,174). The self-reported BMI underestimates the true prevalence of BMI in the SLAN dataset (obesity: 23% measured vs 14% self-report; overweight 58% vs 46% respectively) [37].

The age and sex specific estimates of the impact of the tax on obesity and overweight were used to calculate an overall (unadjusted) population estimate for the impact of the tax on obesity and overweight. This (unadjusted) estimate was then adjusted to allow for the greater prevalence of obesity or overweight when measured compared to that based on self-reported data. This was done by applying the percentage change in the obese and overweight population to the measured baseline prevalence of obesity and overweight to give an estimate of the overall number of people who would be affected by the tax. Absolute estimates of the effect of the tax on the percentage change in the obese population by age and sex were left unadjusted.

#### Irish population

The baseline population by five year age groups and by sex are taken from the 2011 census [38]. The population was split into approximate tertiles based on net household income as measured in the SLAN dataset; they are grouped as follows: group 1 has an annual income of less than €19,999, group 2 has an annual income of between €20,000 and €39,999, and group 3 has an annual income of greater than €40,000. The estimates of overall population consumption were weighted by sex and age (5-year bands).

### Modelling

The 10% tax combined with the pass on rate of 90% was used to estimate the price rise which was then combined with the own-price elasticity estimate of −0.9 to estimate the change in purchasing (and consumption) of SSBs.

#### Changes in calorie consumption

The same percentage change in consumption was applied to all age and income groups. The different baseline consumptions by age, sex and income combined with the percentage change in consumption give different absolute estimates for change in volume consumed by age, sex and income. Food composition tables, specific to the SLAN dataset, were used to estimate the change in calorie consumption based on the change in volume consumed.

#### Change in prevalence of obesity

A comparative risk assessment model (known as PRIME and previously as DIETRON) was used to estimate the changes in the obese and overweight population of Ireland based on age, sex and income specific dietary calorie change, and the underlying age, sex, and income specific BMI of the Irish population. The PRIME model has previously been used to estimate changes in mortality from chronic disease deaths due to potential changes in diet and other health-related behaviours in the UK [39-41].

The equations that the PRIME model uses to estimate change in population BMI were developed by Christiansen and Garby and assume that changes in body weight observe the principles of conservation of energy [42]. Different equations are used for men and women. The equations predict the new ‘steady state’ body weight that is achieved if either total calorie intake or physical activity levels (or both) were to change. They allow for change in the basal metabolic rate and the distribution of fat and lean mass within the body as weight changes. The equations do not predict how long it will take to achieve this new steady state. For the purpose of the modelling conducted here, it was assumed that average physical activity levels remain unchanged.

Estimates of change in obesity and overweight prevalence by income group were weighted according to the age and sex distribution of the Irish population.

#### 95% credible intervals

The 95% credible intervals around estimates of changes in the prevalence of obesity were derived from 5,000 iterations of a Monte Carlo analysis that allow the risk parameters to vary according to the distribution described by Christiansen and Garby [42]. Running the Monte Carlo analysis is time intensive, therefore credible intervals were only calculated for the primary results of the analysis (overall population and income group effects on obesity prevalence), and point estimates only were derived for secondary results (effect on obesity prevalence by age group).

### Sensitivity analysis

A sensitivity analysis was undertaken for two of the assumptions: the tax pass on rate and the price elasticity value. The modelling was repeated firstly assuming a tax pass on rate of 80% and a price elasticity of −0.8 (meaning a 6.4% reduction in consumption of SSBs following a 10% price increase), and secondly assuming a tax pass on rate of 100% and a price elasticity of −1.0 (meaning a 10% reduction in consumption of SSBs).

Further sensitivity analysis was undertaken around the method for estimating how calorie changes predict changes in body weight. The effect of a second set of equations, the Hall and Jordan equations, in predicting changes in body weight for changes in energy intake was considered [27,43]. These equations predict how body weight changes in response to a change in mean daily energy intake, or a change in activity levels. They are based on a simple mathematical model of how diet changes effect body glycogen and body fluids, and how these in turn affect stores of body fat and lean tissue. The equations have been validated based on experimental human feed trials.

Comparisons were made using an online calculator [44] at the individual level; they were made for a hypothetical 30-year old individual who is just overweight (BMI = 26 kg/m^2^) and who is just obese (BMI = 31 kg/m^2^) to illustrate how the respective equations would perform around the cut-offs for overweight and obesity. The estimated mean calorie reduction from the 10% tax was applied to these hypothetical individuals. To show how the performance of the two equations varied as the calorie reduction changes, the effect of a larger calorie reduction on an obese individual (BMI of 31 kg/m^2^) was also modelled. The large calorie changes applied were a reduction of 5 kcal/day (equivalent to the mean reduction for younger groups), and a reduction of 135 kcal/day (equivalent to drinking one less portion of an SSB per day). Comparisons were made for both males and females.

## Results

### Energy intake

We estimate that a 10% tax on SSBs will result in a mean reduction in energy intake of 2.1 kcal/person/day (15 kcal/week, 770 kcal/year) (Table 1). Reductions in calorie intake are greater among the young, and decline with age (Table 1). This reflects patterns of SSB consumption by age (see Additional file 1). The reduction in energy intake is greater for men than and women (Table 1).

**Table 1:** Estimated reduction in energy intake from a 10% SSB tax by age

**Age**	**Mean reduction in daily energy intake (kcal/person/day)**		
	**Female**	**Male**	**Overall**
18-24	3.7 (2.9 to 4.5)	4.7 (3.7 to 5.8)	4.2 (3.3 to 5.2)
25-34	2.7 (2.1 to 3.3)	3.1 (2.4 to 3.8)	2.9 (2.3 to 3.5)
35-44	2.2 (1.7 to 2.7)	2.2 (1.7 to 2.7)	2.2 (1.7 to 2.7)
45-54	1.3 (1.0 to 1.6)	1.6 (1.3 to 2.0)	1.5 (1.1 to 1.8)
55-64	1.0 (0.8 to 1.2)	1.3 (1.0 to 1.6)	1.2 (0.9 to 1.4)
65-74	0.5 (0.4 to 0.7)	1.3 (1.0 to 1.6)	0.9 (0.7 to 1.1)
75+	0.7 (0.6 to 0.9)	0.9 (0.7 to 1.1)	0.8 (0.6 to 1.0)
Overall	1.9 (1.5 to 2.3)	2.3 (1.8 to 2.9)	2.1 (1.7 to 2.6)

Estimates are based on a tax pass on rate of 90%, price elasticity of −0.9 (range is for 80% and −0.8 respectively, and 100% and −1.0). Estimates for each age group are weighted for sex; and the overall estimate is weighted for age and sex of the Irish population.

Reductions in energy intake by income group show different patterns for women and men (Table 2). For women, there is a trend for decreasing reductions in calorie intake with increasing income. For men there is a trend for increasing reductions in calorie intake with increasing income. Both these effects are explained by differences in baseline consumption.

**Table 2:** Estimated reduction in energy intake from a 10% SSB tax by income group

**Income group**	**Mean reduction in daily calorie intake (kcal/person/day)**		
	**Female**	**Male**	**Overall**
Income group 1 (lowest)	2.2	1.6	1.9
Income group 2	1.9	1.9	1.9
Income group 3 (highest)	1.9	2.6	2.3

Incomes groups are based on level of net household income and are grouped as follows: Group 1: <€19,999 per year; Group 2: €20,000-€39,999 per year; Group 3: >€40,000 per year. Estimates are based on a tax pass on rate of 90%, price elasticity of −0.9. Estimates are weighted for age and sex of the Irish population.

### Obesity and overweight

Based on self-reported BMI, the 10% tax is predicted to reduce the number of adults who are obese (BMI ≥ 30 kg/m^2^) by 6,170 (Table 3). The number of adults who are obese or overweight (BMI ≥ 25 kg/m^2^) is predicted to decrease by 11,650. After adjustment for under-estimation for self-reporting, this would equate to 9,900 fewer adults with obesity, and 14,400 fewer adults who are overweight or obese.

**Table 3:** Estimated reduction in obesity from a 10% tax by age (CI = credible interval)

**Age**	**Percentage reduction in obesity (number of people shown in brackets)**		
	**Female**	**Male**	**Overall**
18-24	2.4% (410)	3.3% (350)	2.9% (790)
25-34	2.2% (850)	2.0% (940)	2.1% (1,790)
35-44	1.6% (730)	1.3% (780)	1.4% (1,510)
45-54	0.8% (440)	0.9% (540)	0.8% (980)
55-64	0.7% (290)	0.8% (360)	0.7% (660)
65-74	0.5% (90)	0.7% (200)	0.6% (290)
75+	0.5% (60)	0.7% (50)	0.6% (110)
Overall	1.3% (2,920)	1.2% (3,250)	1.3% (6,170)
	(95% CI: 1,920 to 3,860)	(95% CI: 2,310 to 4,200)	(95% CI: 4,240 to 8,060)
Overall (adjusted)	1.3% (5,310)	1.2% (4,600)	1.3% (9,900)
	(95% CI: 3,490 to 7,020)	(95% CI: 3,260 to 5,920)	(95% CI: 6,750 to 12,940)

Estimates are of the reduction in adults with a BMI ≥ 30 kg/m^2^ and are based on a tax pass on rate of 90%, price elasticity of −0.9. Numbers may not sum due to rounding. Adjusted results are scaled up for under-reporting of obesity; they are derived assuming a baseline prevalence of male obesity of 22% and female obesity of 24% (compared to 16% and 13% respectively in the unadjusted results). Estimates for each age group are weighted for sex; and the overall estimate is weighted for age and sex of the Irish population.

The reductions in obesity are greater in young adults compared to older adults. The greatest relative reduction occurs in those aged 18–24 years, and greatest absolute reduction in those aged 25–34 years (Table 3). The reductions in obesity are similar among men and women (credible intervals overlap). Estimates for overweight are available in Additional file 2.

Reductions in obesity by income show different patterns for men and women. For women the greatest reductions in obesity are seen in the lowest income groups and for men the greatest reductions in obesity are in the highest income group, with a positive trend across the income groups (Table 4). The corresponding estimates for overweight are available in the Additional file 3.

**Table 4:** Estimated impact on obesity of a 10% tax by income (95% credible intervals)

**Income group**	**Percentage reduction in obesity**		
	**Female**	**Male**	**Overall**
Income group 1 (lowest)	1.4% (1.0% to 1.9%)	0.7% (0.5% to 1.0%)	1.1% (0.7% to 1.4%)
Income group 2	1.2% (0.8% to 1.6%)	1.0% (0.7% to 1.3%)	1.1% (0.7% to 1.4%)
Income group 3 (highest)	1.2% (0.8% to 1.6%)	1.5% (1.0% to 1.9%)	1.4% (0.9% to 1.8%)

Estimates are of the reduction in adults with a BMI ≥ 30 kg/m^2^. Income groups are based on level of net household income and are grouped as follows: Group 1: <€19,999 per year; Group 2: €20,000-€39,999 per year; Group 3: >€40,000 per year. Estimates are based on a tax pass on rate of 90% and price elasticity of −0.9. Estimates are weighted for age and sex of the Irish population.

### Sensitivity analyses

Taking a lower value for the tax pass on rate (80%) and the price elasticity (−0.8) (equivalent to a 6.4% reduction in consumption for a 10% tax) the predicted reduction in obesity is 1.0% (6,750 people); and the predicted reduction in overweight, including obese, is 0.5% (9,790 people). Taking a higher value for the tax pass on rate (100%) and the price elasticity (−1.0) (equivalent to a 10% reduction in consumption for a 10% tax) the predicted reduction in obesity is 1.5% (12,940 people); and the predicted reduction in overweight, including obese, is 1.0% (17,820 people).

Comparison of the effect on body weight of the predicted reductions in calorie intake from the 10% tax were similar when using the two different sets of equations (Christiansen and Garby vs Hall and Jordan [21,42]), see Additional files 4 and 5. Only with substantially larger calorie changes do the two equations differ in their outcome, but principally only for women. In these circumstances the Hall and Jordan equation is more conservative in its estimate of weight loss.

## Discussion

We predict that a 10% tax on SSBs in Ireland would result in around 9,900 fewer adults with obesity and around 14,400 fewer adults who are obese or overweight. The greatest reductions would be seen in young to middle-aged adults (25–44 years). The tax appears to have a broadly similar impact, in terms of obesity, across different income groups. This study contributes to discussions of the proposed tax in Ireland using country specific data on population, consumption and body mass index.

### Strengths and limitations

This study has a number of strengths. Firstly it uses a widely published model (PRIME), based on a validated set of equations for how energy intake affects BMI, to estimate how the predicted consumption changes impact on obesity and overweight. Secondly, it explores the differential effects of an SSB tax, both by income and age. Third, it is one of the first studies to explore the effects of an SSB tax outside the US, where patterns of SSB consumption and obesity prevalence may be different [27].

A limitation of this study is that it does not use empirical estimates of own and cross-price elasticities for Ireland. This is because there are no Ireland specific data that could be used. Furthermore, the modelled tax is based on two categories from the SLAN food frequency questionnaire: “fizzy soft drinks” (not low calorie), and “fruit squash”. Within the SLAN dataset, there are no specific categories for energy drinks or fruit juice with added sugar so changes in consumption of these drinks have not been explicitly modelled. Nonetheless the point-estimate of −0.9 for own-price elasticity is more conservative than estimates for Ireland, [5] and similar [14] or less than that used in the US modelling studies reductions [15,22,29]. It is also consistent with a recent meta-analysis estimate of −0.93 [31]. A more recent review from US studies a price elasticity of demand for SSBs of −1.21 (range −0.71 to −3.87), averaged across 12 studies, although the definition here tended to be carbonated drinks with added sugar rather than any soft drink with added sugar [45]. We also note elasticity estimates for sports drinks and fruit juices (both of which form part of the government Special Action Group on Obesity’s definition of SSBs but are not explicitly collected in the SLAN food frequency questionnaire) are relatively large (−2.44 and −1.41 respectively) and greater than the estimates we applied here [45].

While empirical cross-price data exists for other countries, it was not thought appropriate to use such data as the substitution effects (and cross-price effects measured as a percentage change) are likely to be heavily dependent on baseline consumption of the substituted product, which may vary markedly between countries. For this reason using data from the US, or another country, would be highly unreliable. The incorporation of cross-price effects would be likely to attenuate the observed effects. Consistent with this, some animal and human experimental data suggest that sugar consumption is addictive which might suggest that if people consume fewer SSBs, [46] they may seek to increase sugar consumption in other parts of their diet. However, part of the rationale for taxing SSBs is that their consumption leads to ‘passive over-consumption’ , and that it should be possible to reduce the consumption of SSBs without stimulating appetite and causing compensatory consumption of other calories [5,10,24]. The evidence of the relative impact of these two effects is mixed. Some US data suggest that an SSB tax would not lead to a shift towards sugary foods, [47] although other trial data suggest some compensation with food calories may occur [48]. We also note that substitution towards fruit juice and milk in response to an SSB tax, both of which would attenuate the impact of the modelled tax on calorie reduction, have been described [28,47,49].

Although our study provides estimates of impact by age and income group, these are not based on age and income specific price-elasticity estimates. This is because age and income specific estimates are not available for the Irish population. Other studies have found that the price elasticity will vary by income group with some indicating that people in lower income groups will be more price-elastic,[50,51] and others finding higher income groups to be more price-elastic [49,52]. We have also not considered the impact by baseline consumption and there is some data to suggest that high consumers may be less price-elastic than low consumers [47,53]. Given that in Ireland people from lower income groups consume more SSBs, if they were less price-elastic we may be overestimating the effect on population obesity, however, if they were more price elastic our results may be an underestimation.

Price responsiveness by age has not been explored. Differential price responsiveness by age is possible and if the price response was greater among the older population this might attenuate the age pattern we have observed. However given that consumption is so strongly patterned by age (68% of 18–24 year olds drink one serving a month compared to 16% of those aged over 65 years), [25] we feel that it is unlikely that large differences in price responsiveness will occur between different age groups such that the age-effects might disappear. Moreover differential price-responsiveness by age may be a less important factor as some SSBs are brought for consumption in the home (and are not purchased directly by the individual who consumes those drinks).

The pass on rate was assumed to be 90%, in line with reports from Ireland and France [30,54]. However while this assumption may be reasonable there may be differential pass-on rates for different sectors which may impact differently on different groups [32,55]. These effects have not been captured by our work. The tax we modelled was an ad valorem tax, as that was the proposed tax by the Irish minister, and also the typical form of SSB tax introduced [34,56]. However a fixed price rise per unit volume tax has been proposed as a better health tax. This removes an incentive to shift to bulk buying and ensures comparable absolute price increases on cheaper products which may deter people from shifting to cheaper products to maintain consumption [11]. The focus of this paper is the impact on obesity, other health outcomes (e.g. dental caries, type 2 diabetes) have not been considered. Nor have we considered the effect on non-health outcomes, such as disposable income. Like other indirect taxes, this tax is likely to be regressive [34,49,57].

### Comparison with other studies

Most studies examining the effects of an SSB tax have come from the US, where consumption of SSBs is much greater [27,29]. The US studies estimate reductions in energy intake of 7 to 48 kcal per person per day, with lower estimates corresponding to studies that only considered purchases for consumption at home, and the larger estimates considering both purchases for consumption at home and outside the home [29]. Our estimate is similar to an estimate for the UK, which predicted that a 10% tax would reduce consumption by 53 ml/week (equivalent to 3 kcal per person per day) [58]. A recent meta-analysis (n = 3) predicted a 0.02% reduction in energy intake for each 1% price rise on SSBs. For Ireland this would equate to 0.2% reduction in energy intake, or around 4 kcal per person per day [31].

### Policy implications

This study suggests that a tax on SSBs in Ireland would have a small but meaningful effect on obesity. While such a tax would be perceived as affecting the whole population, from a health perspective the tax will predominantly affect younger adults who are the main purchasers and consumers of SSBs (assuming similar price elasticity values across age groups). Although improving the health of young people offers the potential for life-long benefits, immediate health savings from reductions in obesity may not be great, as the complications of obesity like cardiovascular disease and cancer tend to occur in middle to older age.

While the average change in calories may appear small, an average excess energy intake of 100 kcal to 200 kcal per person per day may be sufficient to explain the obesity epidemic [43,59]. Furthermore, a recent meta-analysis has shown that increased sugar consumption is associated with weight gain in adults [60]. An SSB tax is one means of contributing to reversing this excess. It is an example of the prevention paradox: population interventions that aim to improve health have relatively small benefits to the health of most people however the overall positive effect is greater than if only targeting those who drink large quantities of SSBs [61].

Given that SSB consumption in Ireland is relatively low, it may be prudent to consider taxing a broader range of unhealthy food items. However this would be dependent on identifying an appropriate range of unhealthy food items that could be readily taxed, are clearly associated with harm, and have limited potential for unwanted substitution effects. A tax on SSBs should not be seen as a solution in its own right, but should be part of a broader approach to tacking diet-related diseases.

### Unanswered questions and future research

Key areas of uncertainty highlighted during this work are the extent to which any tax rise is passed onto the consumer as a price rise, the nature and extent of any substitution with other beverages, and the time period over which changes in consumption and health occur. How these factors vary between different groups within the population is also unknown. While we have produced estimates of the effect by sex, age and income, it is uncertain whether these factors are true determinants of the responsiveness of consumption to price changes. The response of industry and the extent of public acceptance of an SSB tax are uncertain, and like the full implications of such a tax, these responses may only emerge once a tax is introduced.

## Conclusion

A 10% tax on SSBs in Ireland is estimated to reduce the number of obese adults by around 10,000, and those who are overweight or obese by around 14,000. The greatest effects will be seen in young and middle aged adults, who consume more SSBs.

### Availability of supporting data

The data sets supporting the results of this article are freely available.

The Survey on Lifestyle and Attitude to Nutrition (SLAN) is freely available on request from the Irish Social Science Data Archive, http://www.ucd.ie/issda/.

The 2011 Census data is freely available online from the Irish Central Statistics Office, http://www.cso.ie/en/census/index.html.

## Abbreviations

BMI: Body mass index; IUNA: Irish Universities Nutrition Alliance; SLAN: The survey on lifestyle and attitude to nutrition; SSB: Sugar-sweetened beverage.

## Competing interests

The authors declare they have no competing interests. MR and PS are funded by grant 021/P&C/Core/2010/HPRG by the British Heart Foundation, who had no part in the research design, process, or manuscript preparation.

## Authors’ contributions

DO chaired the steering committee for the health impact assessment of which this research formed a part. MR and DM were members of that committee. All authors contributed to designing the research methods. PS designed the comparative risk assessment model. DM identified the price elasticity data and pass-on rates. AB and OM conducted the modelling. AB and OM drafted the manuscript. All authors contributed to manuscript revisions and approved the final draft.

## Authors’ information

Adam D M Briggs and Oliver T Mytton are joint first author.

## Pre-publication history

The pre-publication history for this paper can be accessed here:

http://www.biomedcentral.com/1471-2458/13/860/prepub

## Supplementary Material

Additional file 1 **The number of calories consumed from SSBs per person per day by age.** Estimates are taken from SLAN 2007. Overall estimates are adjusted for age and sex. Click here for file

Additional file 2 **Estimated reduction in overweight and obesity from a 10% tax by age (CI = credible interval).** Estimate is of the reduction in adults with a BMI ≥ 25 kg/m^2^ and is based on a tax pass on rate of 90%, price elasticity of −0.9. Numbers may not sum due to rounding. Estimates for each age group are weighted for sex; and the overall estimate is weighted for age and sex of the Irish population. Adjusted results are scaled up for under-reporting of overweight and obesity; they are derived assuming a baseline prevalence of male overweight and obesity of 67% and female overweight and obesity of 56% (compared to 59% and 42% respectively in the unadjusted results). Click here for file

Additional file 3 **Percentage reduction in overweight and obesity by income group (95% credible intervals).** Estimates are of the reduction in adults with a BMI ≥ 25 kg/m^2^. Incomes groups are based on level of net household income and are grouped as follows: Group 1: <€19,999 per year; Group 2: €20,000-€39,999 per year; Group 3: >€40,000 per year. Estimates are based on a tax pass on rate of 90% and price elasticity of −0.9. Click here for file

Additional file 4 **Comparison of weight loss predicted for a given calorie change calculated by different methods (females).** A 30 year old female with a BMI of 31 kg/m^2^ is assumed to have a height of 1.63 m and a weight of 82.4 kg; A 30 year old female with a BMI of 26 is assumed to have a height of 1.63 m and a weight of 69.1 kg. Equations compared are Hall and Jordan versus Christiansen and Garby. Click here for file

Additional file 5 **Comparison of weight loss predicted for a given calorie change calculated by different methods (males).** A 30 year old male with a BMI of 31 is assumed to have a height of 1.77 m and a weight of 97.1 kg; A 30 year old male with a BMI of 26 is assumed to have a height of 1.77 m and a weight of 81.5 kg. Equations compared are Hall and Jordan versus Christiansen and Garby. Click here for file
