# Supplementary material for: The potential impact on obesity of a 10% tax on sugar-sweetened beverages in Ireland, an effect assessment modelling study
Source: BMC Public Health. 2013 Sep 17;13:860. doi: 10.1186/1471-2458-13-860 (PMC3852031; doi:10.1186/1471-2458-13-860)
Supplement: Additional file 1 — The number of calories consumed from SSBs per person per day by age. Estimates are taken from SLAN 2007. Overall estimates are adjusted for age and sex. [file 1471-2458-13-860-S1.docx]

**Additional file 1**

**The number of calories consumed from SSBs per person per day by age**

Estimates are taken from SLAN 2007. Overall estimates are adjusted for age and sex.

| Age | Total number of calories consumed from SSBs (kcal/person/day) | | |
| --- | --- | --- | --- |
|  | **Female** | **Male** | **Overall** |
| 18-24 | 45.5 | 58.4 | 51.9 |
| 25-34 | 33.0 | 37.9 | 35.4 |
| 35-44 | 26.8 | 27.1 | 27.0 |
| 45-54 | 15.7 | 20.2 | 18.0 |
| 55-64 | 12.4 | 16.1 | 14.2 |
| 65-74 | 6.7 | 15.6 | 11.1 |
| 75+ | 3.6 | 5.5 | 4.4 |
| Overall | 23.0 | 28.4 | 25.6 |
