# Supplementary material for: The potential impact on obesity of a 10% tax on sugar-sweetened beverages in Ireland, an effect assessment modelling study
Source: BMC Public Health. 2013 Sep 17;13:860. doi: 10.1186/1471-2458-13-860 (PMC3852031; doi:10.1186/1471-2458-13-860)
Supplement: Additional file 2 — Estimated reduction in overweight and obesity from a 10% tax by age (CI = credible interval). Estimate is of the reduction in adults with a BMI ≥ 25 kg/m2 and is based on a tax pass on rate of 90%, price elasticity of −0.9. Numbers may not sum due to rounding. Estimates for each age group are weighted for sex; and the overall estimate is weighted for age and sex of the Irish population. Adjusted results are scaled up for under-reporting of overweight and obesity; they are derived assuming a baseline prevalence of male overweight and obesity of 67% and female overweight and obesity of 56% (compared to 59% and 42% respectively in the unadjusted results). [file 1471-2458-13-860-S2.docx]

**Additional file 2**

**Estimated reduction in overweight and obesity from a 10% tax by age (CI=credible interval)**

Estimate is of the reduction in adults with a BMI ≥ 25kg/m^2^ and is based on a tax pass on rate of 90%, price elasticity of 0.9. Numbers may not sum due to rounding. Estimates for each age group are weighted for sex; and the overall estimate is weighted for age and sex of the Irish population. Adjusted results are scaled up for under-reporting of overweight and obesity; they are derived assuming a baseline prevalence of male overweight and obesity of 67% and female overweight and obesity of 56% (compared to 59% and 42% respectively in the unadjusted results).

| Age | % reduction in overweight including obese (reduction in number) | | |
| --- | --- | --- | --- |
|  | **Female** | **Male** | **Overall** |
| 18-24 | 2.8% (1,320) | 2.7% (1,450) | 2.7% (2,770) |
| 25-34 | 1.4% (1,980) | 0.9% (1,680) | 1.1% (3,660) |
| 35-44 | 1.0% (1,370) | 0.4% (1,010) | 0.6% (2,380) |
| 45-54 | 0.4% (630) | 0.3% (640) | 0.4% (1,270) |
| 55-64 | 0.3% (400) | 0.2% (400) | 0.3% (800) |
| 65-74 | 0.2% (170) | 0.3% (280) | 0.3% (450) |
| 75+ | 0.3% (140) | 0.2% (120) | 0.2% (250) |
| Overall | **0.8% (6,030)**  **(95% CI: 4,040 to 8,020)** | **0.6% (5,630)**  **(95% CI: 3,890 to 7,230)** | **0.7% (11,650)**  **(95% CI: 7,930 to 15,250)** |
| Overall (adjusted) | **0.8% (8,060)**  **(95% CI: 5,390 to 9,640)** | **0.6% (6,320)**  **(95% CI: 4,400 to 8,180)** | **0.7% (14,380)**  **(95% CI: 9,790 to 17,820)** |
