# Supplementary material for: The potential impact on obesity of a 10% tax on sugar-sweetened beverages in Ireland, an effect assessment modelling study
Source: BMC Public Health. 2013 Sep 17;13:860. doi: 10.1186/1471-2458-13-860 (PMC3852031; doi:10.1186/1471-2458-13-860)
Supplement: Additional file 3 — Percentage reduction in overweight and obesity by income group (95% credible intervals). Estimates are of the reduction in adults with a BMI ≥ 25 kg/m2. Incomes groups are based on level of net household income and are grouped as follows: Group 1: <€19,999 per year; Group 2: €20,000-€39,999 per year; Group 3: >€40,000 per year. Estimates are based on a tax pass on rate of 90% and price elasticity of −0.9. [file 1471-2458-13-860-S3.docx]

**Additional file 3**

**Percentage reduction in overweight and obesity by income group** **(95% credible intervals)**

Estimates are of the reduction in adults with a BMI ≥ 25kg/m^2^. Incomes groups are based on level of net household income and are grouped as follows: Group 1: <€19,999 per year; Group 2: €20,000-€39,999 per year; Group 3: >€40,000 per year. Estimates are based on a tax pass on rate of 90% and price elasticity of 0.9.

| Income group | Percentage reduction in obesity | | |
| --- | --- | --- | --- |
|  | **Female** | **Male** | **Overall** |
| Income group 1 (lowest) | 0.6% (0.4% to 0.8%) | 0.4% (0.3% to 0.5%) | 0.8% (0.5% to 1.1%) |
| Income group 2 | 0.6% (0.4% to 0.8%) | 0.5% (0.3% to 0.6%) | 0.8% (0.6% to 1.1%) |
| Income group 3 (highest) | 0.7% (0.5% to 1.0%) | 0.6% (0.4% to 0.8%) | 0.9% (0.6% to 1.2%) |
