# Supplementary material for: The potential impact on obesity of a 10% tax on sugar-sweetened beverages in Ireland, an effect assessment modelling study
Source: BMC Public Health. 2013 Sep 17;13:860. doi: 10.1186/1471-2458-13-860 (PMC3852031; doi:10.1186/1471-2458-13-860)
Supplement: Additional file 4 — Comparison of weight loss predicted for a given calorie change calculated by different methods (females). A 30 year old female with a BMI of 31 kg/m2 is assumed to have a height of 1.63 m and a weight of 82.4 kg; A 30 year old female with a BMI of 26 is assumed to have a height of 1.63 m and a weight of 69.1 kg. Equations compared are Hall and Jordan versus Christiansen and Garby. [file 1471-2458-13-860-S4.docx]

**Additional file 4**

**Comparison of weight loss predicted for a given calorie change calculated by different methods (females)**

A 30 year old female with a BMI of 31 kg/m^2^ is assumed to have a height of 1.63m and a weight of 82.4kg; A 30 year old female with a BMI of 26 is assumed to have a height of 1.63m and a weight of 69.1kg. Equations compared are Hall and Jordan versus Christiansen and Garby.

|  | Weight loss in kg (BMI reduction in kg/m^2^) | |
| --- | --- | --- |
|  | **Hall and Jordan** | **Christiansen and Garby** |
| 30 year old female: BMI of 31  2 kcal/day reduction | 0.1kg  (0.04kg/m^2^) | 0.11kg  (0.04kg/m^2^) |
| 30 year old female: BMI of 26  2 kcal/day reduction | 0.1kg  (0.04kg/m^2^) | 0.11kg  (0.04kg/m^2^) |
| 30 year old female: BMI of 31  5 kcal/day reduction | 0.2kg  (0.09kg/m^2^) | 0.27kg  (0.10kg/m^2^) |
| 30 year old female: BMI of 31  135 kcal/day reduction | 6.4kg  (2.4kg/m^2^) | 7.31kg  (2.75kg/m^2^) |
