# Supplementary material for: The potential impact on obesity of a 10% tax on sugar-sweetened beverages in Ireland, an effect assessment modelling study
Source: BMC Public Health. 2013 Sep 17;13:860. doi: 10.1186/1471-2458-13-860 (PMC3852031; doi:10.1186/1471-2458-13-860)
Supplement: Additional file 5 — Comparison of weight loss predicted for a given calorie change calculated by different methods (males). A 30 year old male with a BMI of 31 is assumed to have a height of 1.77 m and a weight of 97.1 kg; A 30 year old male with a BMI of 26 is assumed to have a height of 1.77 m and a weight of 81.5 kg. Equations compared are Hall and Jordan versus Christiansen and Garby. [file 1471-2458-13-860-S5.docx]

**Additional file 5**

**Comparison of weight loss predicted for a given calorie change calculated by different methods (males)**

A 30 year old male with a BMI of 31 is assumed to have a height of 1.77m and a weight of 97.1kg; A 30 year old male with a BMI of 26 is assumed to have a height of 1.77m and a weight of 81.5kg. Equations compared are Hall and Jordan versus Christiansen and Garby.

|  | Weight loss in kg (BMI reduction in kg/m^2^) | |
| --- | --- | --- |
|  | **Hall and Jordan** | **Christiansen and Garby** |
| 30 year old male: BMI of 31  2kcal/day reduction | 0.1kg  (0.03kg/m^2^) | 0.09kg  (0.03kg/m^2^) |
| 30 year old male: BMI of 26  2 kcal/day reduction | 0.1kg  (0.03kg/m^2^) | 0.09kg  (0.03kg/m^2^) |
| 30 year old male: BMI of 31  5 kcal/day reduction | 0.2kg  (0.07kg/m^2^) | 0.23kg  (0.07kg/m^2^) |
| 30 year old male: BMI of 31  135 kcal/day reduction | 6.1kg  (1.9kg/m^2^) | 6.25kg  (2.00kg/m^2^) |
